# Insect Antimicrobial Peptides, a Mini Review

**DOI:** 10.3390/toxins10110461

**Published:** 2018-11-08

**Authors:** Qinghua Wu, Jiří Patočka, Kamil Kuča

**Affiliations:** 1 College of Life Science, Yangtze University, Jingzhou 434025, China; wqh212@hotmail.com; 2 Department of Chemistry, Faculty of Science, University of Hradec Kralove, 500 03 Hradec Kralove, Czech Republic; 3 Department of Radiology and Toxicology, Faculty of Health and Social Studies, University of South Bohemia, 370 05 Ceske Budejovice, Czech Republic; toxicology@toxicology.cz; 4 Biomedical Research Centre, University Hospital, 500 03 Hradec Kralove, Czech Republic

**Keywords:** antimicrobial peptides, AMP, Structure-activity relationship, modification, mechanism of action

## Abstract

Antimicrobial peptides (AMPs) are crucial effectors of the innate immune system. They provide the first line of defense against a variety of pathogens. AMPs display synergistic effects with conventional antibiotics, and thus present the potential for combined therapies. Insects are extremely resistant to bacterial infections. Insect AMPs are cationic and comprise less than 100 amino acids. These insect peptides exhibit an antimicrobial effect by disrupting the microbial membrane and do not easily allow microbes to develop drug resistance. Currently, membrane mechanisms underlying the antimicrobial effects of AMPs are proposed by different modes: the barrel-stave mode, toroidal-pore, carpet, and disordered toroidal-pore are the typical modes. Positive charge quantity, hydrophobic property and the secondary structure of the peptide are important for the antibacterial activity of AMPs. At present, several structural families of AMPs from insects are known (defensins, cecropins, drosocins, attacins, diptericins, ponericins, metchnikowins, and melittin), but new AMPs are frequently discovered. We reviewed the biological effects of the major insect AMPs. This review will provide further information that facilitates the study of insect AMPs and shed some light on novel microbicides.

## 1. Introduction

Antimicrobial peptides (AMPs) are multifunctional components of the innate immune defense systems in prokaryotic and eukaryotic organisms [1]. Based on amino acid substitutions, AMPs are divided into several subgroups. They generally consist of between 12 and 50 amino acids and are divided into subgroups by their amino acid composition and structure. Some AMPs can be as short as 7 to 100 amino acids [2]. The hydrophobic part of their molecule generally takes up more than 50% of amino acids residues. The secondary structure of AMPs follows four themes: (1) α-helical due to the presence of coiled conformation; (2) β-stranded; (3) β-hairpin or loop; and (4) extended conformation [3].

**Figure 1:**
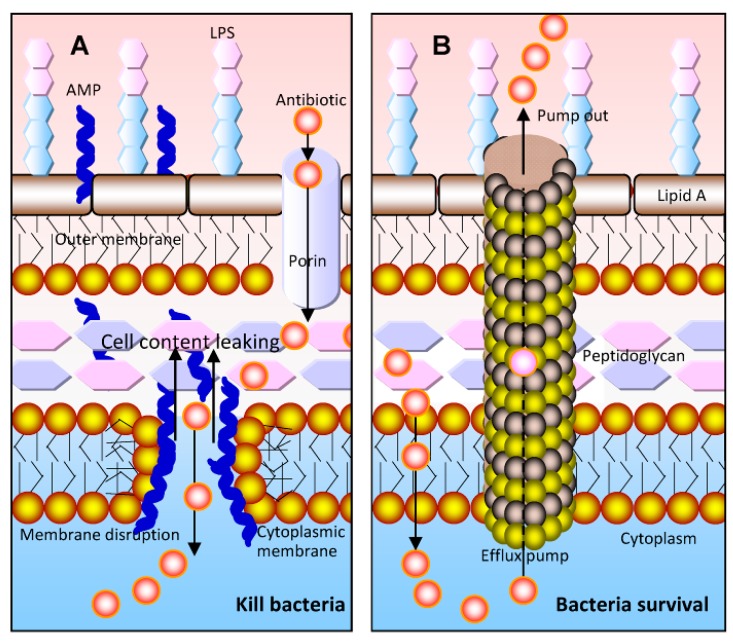
Combined effects of antimicrobial peptides (AMPs) and antibiotics on bacteria. (**A**) AMPs can disrupt the bacterial membrane to cause the leakage of the cell content into the extracellular medium and kill the bacteria. The AMPs can facilitate more antibiotics to enter the cytoplasm of bacteria and finally interact with their target. However, the leakage of the antibiotics from the cytoplasm should not be ignored; (**B**) in bacterial cells, antibiotics are pumped out of the cells by the multidrug efflux pumps, which is how bacteria exert their resistance properties (adapted from [11]).

AMPs have a range of antibacterial, antifungal, and antiviral activities. They have a promising capacity in the therapeutic and prophylactic applications [4,5]. Moreover, AMP-derived drugs are administered as topical formulations to treat skin and wound infections [6]. Some AMPs also show anticancer effects or have anticancer properties [7]. Aurein, for example, is highly effective against around 50 different cancer cell lines and displays little toxicity [8]. Bacteria do not develop resistance to AMPS as easily as to traditional antibiotics. These peptides can physically disrupt microbial cellular membranes and therefore kill a broad spectrum of pathogenic microorganisms. Thus, the microbial membrane is usually considered the primary target of AMPs [9,10]. Moreover, their outstanding membrane disruptive activity makes these peptides ideal candidates for combined therapies with conventional antibiotics [11]. AMPs can facilitate more antibiotic molecules entering the microorganism cytoplasm, where they can interact with their target (Figure 1).

AMPs kill bacteria via a variety of mechanisms including membrane disruption, interference with bacterial metabolism, and targeting of cytoplasmic components [5,6,10,12,13,14]. The primary contact between an AMP and the target bacterium occurs via an electrostatic or hydrophobic interaction, which is strongly dependent on the lipid composition of the bacterial membrane [15,16]. AMPs are capable of interacting with the surface of the cell membrane to alter the permeability of the membrane [10]. After AMPs interact with the cell membrane, the formed transmembrane potential affects the osmotic pressure balance [6]. In short, the interaction between the AMPs and the membrane is directly related to the antibacterial activity of the AMPs. At present, there are at least four modes of action commonly used to describe the membrane activity of AMPs: Barrel-stave, carpet, toroidal-pore, and disordered toroidal-pore [10,12]. For all these modes, a threshold concentration is required to conduct the antibacterial effect [10]. AMPs can also disrupt intracellular enzymes and DNA when they translocate into the pathogens [5]. The detailed explanation of these modes can be read in our recent review [6] as well other publications [5,10,12,13]. Regarding the membrane activity of AMPs, some issues need to be considered. For example, whether there is a specific membrane receptor, and whether there are other factors synergistically working in this context. The mechanism of action of different AMPs may be variable, and further research is needed.

AMPs can be classified into many types, based on their secondary structures in liquid media [17,18]. The β-sheet peptides contain a disulfide bond that stabilizes the structure, and helps the AMPs to cross the cell membrane. In addition to the β-sheet structure, AMPs also form an α-helical structure, and contain a cysteine in the peptide to form an intramolecular disulfide bridge [19,20]. Due to the presence of hydrophobic groups, the peptide chain forms a polymer by hydrophobic interaction to increase the affinity for cell membranes [6,21,22,23]. The optimum antibacterial activity appears to be a balance between charge density, hydrophobic character, and polymer chain length [24,25,26]. Increasing the number of positively charged amino acids or changing their position in the peptide chain can affect the secondary structure of the AMPs, thereby further affecting their antibacterial activity. Thus, the combination of charge, hydrophobicity, and length of the peptide is important for the antimicrobial activity of AMPs [27,28,29].

It is well known that insects are extremely resistant to bacterial infections. They can produce a wide range of proteins and peptides as a first line of defense against pathogen infection [14]. Insects activate immune systems, or directly target bacteria and viruses, to combat pathogens. We have previously reviewed the chemical and biological properties of marine AMPs [6]. In this review, we present characteristic and potential medical applications of insect origin peptides with antimicrobial activity. We especially focus on a large group of AMPs that are present both in ancient and recent insects: Defensins, cecropins, attacins, lebocins, dipterins, ponericins, jelleines, and others. By so doing, we will provide a new perspective on the function and biological effects of insect AMPs as well as their use in medicine.

## 2. Insect Antimicrobial Peptides

Insect AMPs are divided into three groups based on their amino acid sequence and structures: (a) Cecropins, the linear peptides with α-helix but lack cysteine residues; (b) Defensins with 6–8 conserved cysteine residues, have a stabilizing array of 3 or 4 disulfide bridges and 3 domains consisting in a flexible amino-terminal loop; and (c) peptides with an overrepresentation of Proline and/or Glycine residues [30]. The most explored insect AMPs are cecropins, drosocin, attacins, diptericins, defensins, ponericins, drosomycin and metchnikowin. However, more new peptides can still be discovered [31,32]. Most glycine-rich and proline-rich peptides are active against Gram-negative strains of bacteria [33]. Defensins can selectively kill Gram-positive bacteria, whereas cecropins are active against both types [12]. Iinsect AMPs are very potent since their IC_50_ ranges in the submicromolar or low micromolar range. Currently, there are still no insect-derived AMPs on the market yet. However, we have no doubt that insect AMPs can be exploited as an alternative to antibiotics [12].

### 2.1. Defensins

Defensins are a family of small, variable cationic arginine-rich peptides [34]. They are not specific to insects, and more than 300 defensins have been identified so far. Defensin peptides are ancient natural antibiotics with strong antimicrobial activity against a range of microorganisms [35,36]. They consist of 18–45 amino acids with 6–8 conserved cysteine residues [36]. Classic defensins (α-defensins) contain 29–35 amino acids, and the insect defensins contain 29–34 amino acids. The molecule of defensin is usually stabilized by three disulfide bonds and, a β-hairpin is their major structural feature [37]. Defensins bind to the cell membrane or form pore-like membrane defects to efflux of essential ions and nutrients [38,39].

Insect defensins are inducible antibacterial peptides with activity against both Gram-positive and Gram-negative bacteria [40]. They are highly effective against Gram-positive bacteria [41], including human pathogenic bacteria such as *Staphylococcus aureus*. However, these peptides are less effective against Gram-negative bacteria [42]. Insect defensins are isolated from insect orders such as Diptera, Hymenoptera, Coleoptera, Trichoptera, Hemiptera, and Odonata [1,40]. All types of AMPs are reported in lepidopteran insects, except for the insect defensins [43]. Defensin from rabbit neutrophils exerts potent bactericidal activity against the multi-drug-resistant (MDR) strains of *Pseudomonas aeruginosa* [44].

Royalisin is isolated from the royal jelly of *Apis mellifera*. It consists of 51 amino acids (VTCDLLSFKQVNDSACAANCLSLGKAGGHCEKGVCICRKTSFKDLWDKYF-NH_2_), in which six cysteine residues form three disulfide bonds to make the molecule a compact globular structure [45]. Royalisin is an amphipathic protein, and its C-terminal is rich in charged amino acids. This peptide inhibits the Gram-positive bacteria and fungi. It is particularly effective against the larvae of the bee pathogen *Paenibacillus larvae*, which causes American foulbrood [46].

### 2.2. Cecropins

Cecropins were first isolated from the hemolymph of the giant silk moth *Hyalophora cecropia* (cecropia moth), whence the term cecropin was derived [47]. These peptides are mainly structured by a large number of antibacterial and toxic peptides isolated from various lepidopteran and dipteran species, which constitute a major part of the cell-free immunity of insects. Cecropins are small proteins (around 35 amino acid residues) with activity against both Gram-positive and ram-negative bacteria. The principle insect cecropins (A, B and D) consist of 35–37 residues without cysteine [48,49]. Cecropins can lyse bacterial cellular membranes and can also inhibit proline uptake as well as cause leaky membranes [50,51]. Insect cecropins also have other names including bactericidin, lepidopteran, sarcotoxin, etc. [52]. These structurally related peptides are shown in Table 1.

Cecropin A is an AMP with a stabilized α-helical structure [55]. The precise antibacterial mechanism of cecropin A is unclear, but there is primary evidence showing that the cell membrane is the target [15]. Based on recent results, Yun and Lee [56] confirmed that an ion imbalance regulates cecropin A-induced apoptotic activity. Cecropin A can significantly reduce NADPH and glutathione levels to further induce oxidative stress by forming reactive oxygen species (ROS) [56,57]. Initially, cecropin peptides are arranged as antiparallel dimers with conserved residues of adjacent monomers in contact. The dimers may bind to the membrane with the NH_2_-terminal helices sunken into the head-group layer [57]. Cecropin A has promising activity against the fungus *Beauveria bassiana* in silkworm larvae [58].

Cecropin B is a naturally occurring linear cationic peptide consisting of 35 amino acids [59]. It is the member of the cecropin family with the highest antibacterial activity [59]. In a rat mode of septic shock, cecropin B significantly reduced the lethality of *Escherichia coli* load and plasma endotoxin levels [60]. Cecropin B attenuates the motility of the adult female nematode worm *Brugia pahangi* in adult females of *Aedes aegypti* and causes a significant decrease in the number of developing larvae [61]. Cecropin B also shows an antifungal capacity against *Candida albicans* [62]. Cecropin B, as well as other AMPs from the silkworm *Bombyx mori* including moricin (42 amino acids) [63,64], have a broad activity against porcine bacterial pathogens and is quite crucial in the porcine industry [65].

Cecropin C is present in very low quantities in the hemolymph of *H. cecropia.* Currently, the antibacterial activity of cecropin C is rarely reported. Compared with cecropin A, no C-terminal blocking group is present in cecropin C. Cecropin C is considered a precursor or degradation product of cecropin A [48].

Cecropin D is isolated from *H. Cecropia* and shows homology to cecropin A and cecropin B [48]. After bacterial infection, cecropin D appears in the hemolymph later than cecropin A or cecropin B [66]. A recombinant cecropin D has been successfully expressed in *Pichia pastoris* and showed antibacterial activity for both Gram-positive and Gram-negative bacteria [67]. The C-terminal lysine residue of cecropin D could increase antibacterial activity due to activated phosphorylation [68]. Cecropin D also inhibits porcine reproductive and respiratory syndrome virus (PRRSV) infection and replication in vitro [69].

Cecropin P1 is an antibacterial peptide from *Ascaris suum*, a parasitic nematode that resides in pig intestine [54]. Cecropin P1 could effectively inhibit the growth of enterotoxigenic *E. coli* with the minimal inhibitory concentration (MIC) of 1 mg/mL [70]. A tertiary structure study shows that cecropin P1 can form α-helical structures with the C-terminal region (Lys15–Gly29) in lipopolysaccharide (LPS) of the outer membrane of Gram-negative bacteria [71]. The concentration-dependent killing of *E. coli* by cecropin P1 can be driven through the extent of the immediate permeabilizing action of the peptide [72]. Cysteine-terminus modified cecropin P1 (CP1C) shows less antimicrobial activity, since the presence of polyethylene glycol (PEG) linker prevents CP1C from interacting with the bilayer [73]. Cecropin P1 also shows a significant inhibitory effect on human fungal pathogen *C. albicans* [74]. Cecropin P1 inhibits PRRSV by blocking attachment [75]. Cecropin P1 inhibits viral particle release and attenuates virus-induced apoptosis [75]. Currently, biosensors using cecropin P1 have been developed, and the peptide has been immobilized through different termini results in different functions and activities [76].

Lucilin, a 36-residue cecropin, is identified as a partial genetic sequence in *Lucilia sericata* maggots [77]. The fusion protein, GWLK-Lucilin-CPD-His8, shows a potential activity against multidrug resistant (MDR) bacteria *E. coli* [77]. *Musca domestica* cecropin is also a potential bactericidal agent against clinical isolates of *E. coli* [78].

### 2.3. Attacins

**Figure 2:**
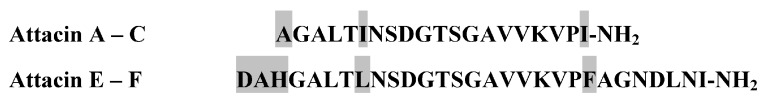
Amino-terminal sequence of attacins A–F. Their sequence difference can be observed from the highlighted residues.

Attacins are glycine-rich proteins, belonging to the AMP group. Attacins were first discovered in *Hyalophora cecropia* [79]. They are effective against Gram-negative bacteria [80]. Attacins A–F are closely related antibacterial proteins, which are isolated from the hemolymph of immunized pupae of the cecropia moth (*Hyalophora cecropia*) [79]. They are a rather heterogeneous group of proteins, varying in size but rich in glycine residues (10–22%). Attacins A–F can be divided into two groups based on their amino acid composition: Attacins A–D constitute a basic group; and attacins E and F, which have acidic residues. Within each group, the forms are very similar (Figure 2).

Attacins act by blocking the synthesis of the major outer membrane proteins in dividing Gram-negative bacteria, thus disturbing the integrity of the cell wall and causing the bacteria to grow in long chains [81]. Attacins constitute an antibacterial active form of inducible immune protein P5. Attacins can effectively kill *E. coli* and other Gram-negative bacteria. In addition to cecropin and lysozyme, attacins are the third antibacterial protein in the humoral immune system of *H. cecropia* [82]. Some attacin and attacin-related proteins are isolated from *Bombyx mori*, *Glossina morsitans* (tse-tse fly), *Heliothis virescens*, *Trichoplusia ni*, *Samia cynthia ricini* (wild silkmoth) and *Musca domestica* (housefly) [83,84].

### 2.4. Lebocins

Lebocins are antibacterial peptides consisting of 32 amino acids, which were identified by Hara and Yamakawa [63] in the hemolymph of the silkworm *Bombyx mori*, immunized with *E. coli* [63]. Lebocin is a proline-rich and O-glycosylated peptide [85]. In total, 41% of the amino acid sequence of lebocin is identical with abaecin, a major 34 amino acid antibacterial peptide (YVPLPNVPQPGRRPFPTFPGQGPFNPKIKWPQGY-NH_2_) in the honeybee *Apis mellifera* [86]. The amino acid sequence in lebocin 1 is DLRFLYPRGKLPVPTPPPFNPKPIYIDMGNRY-NH_2_. The primary sequence of lebocins 1 and 2 differ only in their sugar moiety. Lebocin 3 has the same structure as lebocin 2, except that residue 16 is leucine instead of proline [87].

### 2.5. Drosocin

Drosocin is a peptide produced by *Drosophila melanogaster* [88]. This peptide contains 19 amino acids (GKPRPYSPRPTSHPRPIRV-NH_2_). The peptide is O-glycosylated, and this modification is required for maximum biological activity [89]. Glycosylation is an important post-translational modification for some proline-riched AMPs class [90]. In addition to the Gram-positive bacterium *M. luteus*, Drosocin is primarily resistant to Gram-negative bacteria. Deletion of the first five N-terminal residues completely abolishes the activity of drosocin [88,91]. Glycosylated drosocin is active against *E. coli* and fungi [92].

Apidaecin IB and drosocin show significant sequence homology and interaction mechanism but lack any pore-forming activity [93]. Apidaecins are the major components of the honeybee humoral defence against microbial invasion [93]. N-terminal mutation of apidaecins not only reinforces the interaction with unidentified intracellular target(s), but also promotes the cell-penetration efficiency [94]. Structure N-terminal Ile-Orn- and Trp-Orn-motif repeats increases the antimicrobial activity against *Pseudomonas aeruginosa* [95].

### 2.6. Diptericins

Diptericins constitute a family of related glycine-rich antibacterial peptides (about 8 kD) from *Dipteran* hemolymph proteins of about 82 amino acids [96]. Diptericins A–C have been isolated from immunized larvae of the dipteran *Phormia terranovae* [97]. Diptericin is also expressed in *D. melanogaster* [98], *Sarcophaga peregrina* (flesh fly) [99], and *Mayetiola destructor* (Hessian fly). The predominant member of this family of peptides is diptericin A (DDMTMKPTPPPQYPLNLQGGGGGQSGDGFGFAVQGHQKVWTSDNGRHEIGLNGGYGQHLGGPYGNSEPSWKVGSTYTYRFPNF-NH_2_). This peptide is active only against a limited number of Gram-negative bacteria (*E. coli* K12, *Erwinia hericola T*, and *Erwinia carotovora* 113). Diptericin A mainly acts on the cytoplasmic membrane of growing bacteria [100].

Prolixicin is a novel AMP from the family of diptericins. This peptide has been isolated from the hemipteran insect *Rhodnius prolixus*. Prolixicin is a peptide with 21 amino acids [101]. It has also two putative phosphorylation sites, but no glycosylation sites have been identified. Sequence analysis reveals that one region of prolixicin is related to the diptericin/attacin family of AMPs. This peptide can be produced by midgut tissues after the bacterial infection of the hemolymph [101].

### 2.7. Metchnikowin

In 1995, Russian scientists identified a novel 26-residue proline-rich immune-inducible linear peptide (HRHEGPIFNTRPSPFNPNEPRPGPIY-NH_2_) from *D. melanogaster*, which was remarkable in its unusual antimicrobial activity against Gram-positive bacteria and fungi. However, this peptide has no effect against Gram-negative bacteria [102]. This peptide was named Metchnikowin in honour of E. Metchnikow who initiated this field of research. There are two isoforms of Metchnikowin in the Oregon *Drosophila* strain, which differ by one residue (His compared to Arg) [103]. The target of Metchnikowin has been studied. Metchnikowin interacts with the fungal enzyme β(1,3)-glucanosyltransferase Gel1 (FgBGT), an important enzyme that is involved in fungal cell wall synthesis [104]. Metchnikowin also targets the iron-sulfur subunit (SdhB) of succinate-coenzyme Q reductase (SQR). In the study of Moghaddam et al. [104], Metchnikowin inhibited the SDH activity of *Fusarium graminearum* mitochondrial SQR by up to 52%.

### 2.8. Ponericins

Ponericins are peptides isolated from the venom of the predatory ant *Pachycondyla goeldii*. Members of the subfamily Ponerinae were isolated and their amino acid sequences were characterized by Orivel and coworkers [105]. Ponericins can be classified into three families based on their primary structure similarities: Ponericins G, W, and L (Table 2). Ponericins have high sequence similarities for the known peptides: Ponericin G has similarity with cecropin-like peptides; ponericin W is similar with gaegurins and melittin; and ponericin L is similar with dermaseptins. Ponericins also show hemolytic activities, as well as insecticidal activities against cricket larvae. Ponericins have α-helical structures in cell membranes [106].

### 2.9. Jelleines

Jelleines are a family of peptides isolated from *Apis mellifera* royal jelly [107]. They are composed of 8–9 amino acids and bear a +2 charge at the C-terminus [107]. Four AMPs were purified from royal jelly of honeybees: Jelleine-I (PFKLSLHL-NH_2_), jelleine-II (TPFKLSLHL-NH_2_), jelleine-III (EPFKLSLHL-NH_2_) and jelleine-IV (TPFKLSLH-NH_2_) [108]. Jelleines-I–III presented antimicrobial activities against yeast, fungi, Gram-positive and Gram-negative bacteria [108,109]. These peptides have no similarities with other AMPs from honeybees. Molecules of jelleines are still in the characterization phase [110].

### 2.10. Apisimin

Apisimin is a peptide consisting of 54 amino acids with a primary sequence of KTSISVKGESNVDVVSQINSLVSSIVSGANVSAVLLAQTLVNILQILIDANVFA-NH_2_, and is found in honeybee royal jelly [111], which stimulates the proliferation of human monocytes [112]. Apisimin is rich in valine and serine, and contains only one aromatic amino acid, phenylalanine [108]. Apisimin is a small peptide in royal jelly. High levels of small mRNA expression of apisimin are observed in the heads of nurse and foraging honeybees. Therefore, they may play a physiological role in honeybee colonies [111]. The study of Gannabathula et al. [113] provides evidence that apisimin and arabinogalactan proteins are present in honey and contribute to their immune active properties.

### 2.11. Pyrrhocoricin

Pyrrhocoricin, a proline-rich inducible AMP, was isolated from the hemolymph of the sap-sucking bug *Pyrrhocoris apterus* by Cociancich et al. [114]. This 20 amino acid peptide (VDKGSYLPRPTPPRPIYNRN-NH_2_) interacts with the heat shock protein DnaK, which is correlated with the antimicrobial activity [115]. Pyrrhocoricin can bind to and promote the ATPase activity of the molecular chaperone DnaK [116,117]. Boxell et al. [118] showed that pyrrhocoricin could act as a delivery vehicle in transducing peptides across the cell membrane of the parasite *Cryptosporidium parvum*. The successful transduction facilitates target validation. It will also help to deliver peptide-based drugs to this important human pathogen. Cyclization of pyrrhocoricin structural elements is important for the antimicrobial activity of the native peptide [119].

### 2.12. Persulcatusin

Persulcatusin has been identified in the midgut of *Ixodes persulcatus*. Its amino acid sequence is GFGCPFNQGACHRHCRSIGRRGGYCAGLFKQTCTCYSR-NH_2_ [120]. The complete structure of persulcatusin has not been identified yet. Its similarity with other known tick AMPs is from 71% to 88% [121,122]. The structural integrity of persulcatusin is maintained by three S-S bonds, which are energetically important for the stability and the formation of the structure of α-helix and β-sheet [123,124]. This peptide can inhibit the growth of methicillin-sensitive *S. aureus* (MSSA) and methicillin-resistant *S. aureus* (MRSA) with the MIC of 0.156–1.25 μg/mL and 0.625–2.5 μg/mL, respectively [31]. Very recently, persulcatusin exhibits strong antibacterial activity against MDR *S. aureus* strains, including VRSA [123]. The antimicrobial activity of persulcatusin against MRSA was stronger than that of other AMPs [124].

### 2.13. Melittin

Melittin is a peptide toxin found in bee venom and is effective against bacteria [125,126]. This peptide has a linear structure with 26 amino acid residues (GIGAVLKVLTTGLPALISWIKRKRQQ-NH_2_) [125,127]. Melittin has a strong antibacterial effect against a variety of bacteria including *Borrelia burgdorferi* [128], *Listeria monocytogenes* [129], *S. aureus,* and *P. aeruginosa* [130,131]. Melittin has antibacterial activity against *Xanthomonas oryzae* pv. *oryzae*, a destructive bacterial disease of rice, indicating that this peptide may have potential applications in plant protection [132]. RV-23, a melittin-related peptide, shows strong antibacterial activity against *E. coli* and *S. aureus* [133]. A new synthetic peptide, MelP5, is a gain-of-function variant of melittin [134]. Moreover, this peptide facilitates the passage of macromolecules across bilayers.

Melittin binds to membrane surfaces with a negative charge to disturb the integrity of phospholipid bilayers by forming pores, which subsequently induces the leakage of atomic ions and molecules and ultimately leads to cell lysis [135]. Proline residue is important in the antimicrobial activity of melittin [136]. Analogues lacking the poline residue and dimers decrease the cytotoxicity and minimize the inhibitor concentrations. However, there are ongoing debates regarding the molecular mechanism of melittin [137,138]. For decades, the equilibrium transmembrane pore mode has been considered the major mechanism of the antibacterial activity of melittin. However, emerging evidence shows that the transmembrane pore is not required in this context [139]. Notably, the mechanisms can be markedly influenced by experimental design in different studies [137]. Lee and Lee [140] further reported that melittin triggers apoptosis in *C. albicans* through the ROS-mediated mitochondria and caspase pathway.

Very recently, Akbari et al. [141] showed that there are highly synergistic effects of melittin with conventional antibiotics against MDR isolates of *Acinetobacter baumannii* and *P. aeruginosa*. In their study, the geometric means of MIC for melittin and doripenem after combination were reduced to 61.5- and 51.5-fold, respectively, against *A. baumannii* isolates. This working group [142] further showed that the new melittin-derived peptides MDP1 and MDP2 exhibited efficient antibacterial activity against MDR *S. aureus*, *E. coli*, and *P. aeruginosa*. Melittin also exhibits very effective antibacterial activity against MRSA strains [143]. MRSA-infected mice treated with melittin were successfully rescued from bacteraemia. The clinical application of melittin still needs a lot of work in the future, since most current work is in the preclinical phase [144].

## 3. Concluding Remarks

Insect AMPs are the main immune effector molecules. Therefore, there are large numbers of AMP resources in the huge insect world. In recent years, fruit fly and mosquito have been used as model organisms, and great progress has been made in the study of the natural immunity of insects. There is growing evidence that the natural immune system of insects is much more complex than one might expect. In the meantime, due to the conservation of biological evolution, certain molecules and signaling pathways in the natural immune system of insects have certain similarities with vertebrates (including humans). Studies of the natural immune system of insects helps to further understand the complexity of the human immune system. Currently, more bacteria have developed multidrug resistance due to the abuse of antibiotics, and some super antibiotic resistant bacteria have emerged, which pose a great threat to human health [145]. Finding and developing new antimicrobial drugs has become an urgent problem in the medical field.

AMPs not only have a broad-spectrum killing effect on bacteria and fungi, but also have a killing effect on viruses, protozoa and cancer cells. Compared with traditional antibiotics, their mechanism of action is unique, and it is not easy for AMPs to cause microbial resistance. Most of them do not damage or destroy normal cells of higher animals. For example, very recently, clavaspirin peptide, a peptide from tunicate *Styela clava*, exhibited the capacity to kill drug-resistant pathogens (*S. aureus*) without detectable resistance [146]. The above advantages and rich resource content of insect AMPs make them excellent templates for the development of new antimicrobial drugs. Indeed, some synergistic effects of AMPs with conventional antibiotics are observed against bacteria [141]. However, quantitative methods are rarely used to test this synergistic profile. Wu et al. [147] recently tested the synergistic effect of AMP DP7 and antibiotics on MDRs (*S. aureus*, *E. coli*) using quantitative polymerase chain reaction. Other researchers [148] also tested the synergistic antimicrobial activity of frog peptides via oriented circular dichroism and quantitative solid-state F-19-NMR analysis. In the future, more quantitative studies on the synergistic effects of AMPs with antibiotics should be encouraged. Currently, the market for peptide drugs is increasing steadily, and some products such as Bacitracin, polymyxin and Fuzeon are already on the market [5]. However, the clinical use of AMPs is still limited by some shortcomings, such as low bioavailability, potential hemolysis, instability to proteases, and unknown toxicity [10]. Investigations of natural peptides and nano-delivery systems from natural polymers are a new research focus area for the future [149]. However, there is one issue that needs to be considered: Despite intensive studies, we still do not fully understand the structure-activity relationship (SAR) and mechanisms underlying AMP activity. Therefore, more SAR studies of AMPs are required. Moreover, further investigations into the cellular and molecular mechanisms of AMP effects are warranted. Finally, a library of insect AMPs should be established in order to optimize them further and improve their antimicrobial activity and toxic properties.

## Figures and Tables

**Table 1:** The amino acid sequences of cecropins.

Name	Amino Acid Sequence	Reference
Cecropin A	GGLKKLGKKLEGVGKRVFKASEKALPVAVGIKALG-NH_2_	[47]
Cecropin B	KWKVFKKIEKMGRNIRNGIVKAGPAIAVLGEAKAL-NH_2_	[47]
Cecropin B1	KWKVFKKIEKMGRNIRNGIVKAGPKWKVFKKIEK-NH_2_	[53]
Cecropin B3	AIAVLGEAKALMGRNIRNGIVKAGPAIAVLGEAKAL-NH_2_	[53]
Cecropin C	GWLKKLGKRIERIGQHTRDATIQGLGIAQQAANVAATAR-NH_2_	[48]
Cecropin D	WNPFKELEKVGQRVRDAVISAGPAVATVAQATALAK-NH_2_	[48]
Cecropin P1	SWLSKTAKKLENSAKKRISEGIAIAIQGGPR-NH_2_	[54]

**Table 2:** The amino acid sequences of ponericins, which are antibacterial insect peptides (According to [106]).

Name	Amino Acid Sequence
Ponericin G1	GWKDWAKKAGGWLKKKGPGMAKAALKAAMQ-NH_2_
Ponericin G2	GWKDWLKKGKEWLKAKGPGIVKAALQAATQ-NH_2_
Ponericin G3	GWKDWLNKGKEWLKKKGPGIMKAALKAATQ-NH_2_
Ponericin G4	DFKDWMKTAGEWLKKKGPGILKAAMAAAT-NH_2_
Ponericin G5	GLKDWVKIAGGWLKKGPGILKAAMAAATQ-NH_2_
Ponericin G6	GLVDVLGKVGGLIKKLLP-NH_2_
Ponericin G7	GLVDVLGKVG GLIKKLLPG-NH_2_
Ponericin W1	WLGSALKIGAKLLPSVVGLFKKKKQ-NH_2_
Ponericin W2	WLGSALKIGAKLLPSVVGLFQKKKK-NH_2_
Ponericin W3	GIWGTLAKIGIKAVPRVISMLKKKKQ-NH_2_
Ponericin W4	GIWGTALKWGVKLLPKLVGMAQTKKQ-NH_2_
Ponericin W5	FWGALIKGAAKLIPSVVGLFKKKQ-NH_2_
Ponericin W6	FIGTALGIASAIPAIVKLFK-NH_2_
Ponericin L1	LLKELWTKMKGAGKAVLGKI-NH_2_
Ponericin L2	LLKELWTKIKGAGKAVLGKIKGLL-NH_2_
